# Cortex death precedes stele failure in soybean fine roots during drought but does not prevent plant rehydration

**DOI:** 10.1093/plphys/kiag132

**Published:** 2026-03-13

**Authors:** Beatrice L Harrison Day, Christopher McCarthy, Timothy J Brodribb, Madeline R Carins Murphy, Craig R Brodersen

**Affiliations:** School of the Environment, Yale University, New Haven, CT 06520, United States; Hawkesbury Institute for the Environment, Western Sydney University, Richmond, NSW 2753, Australia; School of Natural Sciences, University of Tasmania, Tas, Hobart 7001, Australia; School of Natural Sciences, University of Tasmania, Tas, Hobart 7001, Australia; School of Natural Sciences, University of Tasmania, Tas, Hobart 7001, Australia; School of the Environment, Yale University, New Haven, CT 06520, United States

## Abstract

Fine roots regulate plant water uptake, but the dynamic cell-level hydraulic behavior of these organs remains poorly understood, particularly at the onset of drought. We investigated changes in fine root (<2-mm diameter) shrinkage, turgor loss, cell layer viability, and uptake during dehydration and rehydration in soybean (*Glycine max*) to identify critical physiological thresholds of water potentials experienced by plants exposed to experimental drought. Fine root diameter shrank by over 50% at the completion of drying, with 55.36% of this relative shrinkage occurring by −0.25 MPa, and most of that water volume loss was attributed to epidermal and cortex cells. Epidermal cells lost turgor at −0.5 MPa and cortex cells reached mortality by −1.0 MPa, prior to xylem embolism onset. Cells within the stele remained viable after cortex and epidermal mortality until −1.75 MPa, coinciding with the 50% loss of xylem conductivity through embolism (whole-plant *P_50_*). Drought recovery experiments revealed that cortical and epidermal cell mortality slowed but did not prevent rehydration of those same cell layers, or whole plant rehydration, prior to embolism. The rapid, dynamic changes in cortical and epidermal cells during the earliest stages of drought exposure and subsequent recovery are likely to act to physically decouple fine roots from the surrounding soil to limit plant dehydration, an effect likely accelerated by bare-root lab drying conditions. Movement of water through these dead cell layers allows rehydration of living stele tissue prior to embolism, supporting root growth and recovery post-drought.

## Teaser

Soybean fine roots begin shrinking early during drought, corresponding with the death of external cortex cells, yet protected inner stele cells survive, supporting rehydration after water returns.

## Introduction

Fine roots <2-mm diameter dominate root networks, comprising 85% to 90% of total root surface area in woody species (Jackson et al. 1997) and almost the entirety of the network area in herbaceous species (Himmelbauer et al. 2004). These fine roots acquire the bulk of water for the plant, with absorptive function performed by the peripheral fine root branching orders (Guo et al. 2008). Consequently, understanding the hydraulic physiology of this root cohort during water deficit has direct relevance for determining precisely when and how a plant will become isolated from soil water. In recent decades, emphasis has been placed on understanding the environmental conditions that cause decreases in productivity and ultimately drought-induced mortality of stems and leaves (Choat et al. 2012; Brodribb et al. 2020). Compared with above-ground hydraulic vulnerability and water use efficiency traits (McAdam and Brodribb 2015; Blackman 2018; Brodersen et al. 2019), the dynamic physiological behavior of root networks spanning the complete dehydration gradient from incipient dehydration to mortality remain comparatively poorly characterized (Peters et al. 2020; Bourbia et al. 2021).

Evidence suggests that much of the resistance to water transport within the plant occurs in the root system (Rodriguez-Dominguez and Brodribb 2020) and primarily within the radial pathway across the root epidermis, cortex, and endodermis (Rieger and Litvin 1999). Contrary to long-held assumptions that the xylem of roots is highly vulnerable to drought-induced embolism, roots appear to be as resistant or more so than xylem in stems, petioles, reproductive structures, and leaves (Skelton et al. 2017; Rodriguez-Dominguez et al. 2018), highlighting that root embolism may not be the immediate determinant of the loss of hydraulic conductivity during drought (Harrison Day et al. 2024). Instead, factors outside of the xylem, such as cortical cell physiology, likely define the resistance to water transport within the roots during dehydration (North and Nobel 1991). During the initial stages of soil moisture deficit, fine roots undergo significant reductions in total volume that physically decouples roots, and therefore the entire plant, from the surrounding soil. This includes the formation of cortical lacunae and cortex cell collapse (Cuneo et al. 2016; Duddek et al. 2022) that results from localized cell desiccation within this tissue layer. Importantly, these volumetric changes occur very early on during drought as water potentials approach ca. −0.5 MPa (Harrison Day et al. 2026), well before the initiation of stomatal closure or xylem embolism (Choat et al. 2012).

Recently Harrison Day et al. (2026) showed that fine roots of phylogenetically diverse species experienced significant shrinkage extremely early during water deficit, where ca. 40% of the total shrinkage occurred under extremely mild water stress conditions (by ca. −0.1 MPa), and most shrinkage was complete prior to the onset of hydraulic failure in the xylem due to embolism. This change in fine root volume was observed across herbaceous and woody plants, suggesting that the pattern is highly conserved and therefore of significant importance for understanding drought physiology. Loss of fine root volume during the early stages of drought is likely driven by a rapid loss of cell turgor as water exits cortical and epidermal cells (Faiz and Weatherley 1982), which leads to the formation of cortical lacunae (Cuneo et al. 2016), air spaces that result from the physical disintegration of cell wall boundaries in the cortex. Here, a key knowledge gap remains between the detailed research documenting root-soil interactions (Carminati et al. 2010; Carminati and Javaux 2020) and the “downstream” research documenting hydraulic failure through embolism (Peters et al. 2020). The potential for the root cells to recover from shrinkage and turgor loss, their survival post-rehydration, and their role as a capacitive reservoir (McCarthy et al. 2025) for water storage provides a direct avenue for understanding plant persistence and recovery post-drought.

Although root turgor loss appears to be an essential component of understanding fine root shrinkage dynamics during drying (Huang and Fry 1998; Duddek et al. 2022), direct measurement of cell turgor is technically challenging and limited in throughput, most often employing the cell pressure probe method (Tomos and Leigh 1999). The recent development of cavitation bubble manometry (CBM) by Brodersen et al. (2025) provides unprecedented, in situ access to changes in plant cell turgor pressure, whereby a microbubble (<10 µm in diameter) is nucleated inside the target cell by vaporizing the cellular contents with a pulse of light. The pressure of the surrounding fluid constrains the maximum microbubble radius, and by studying the dynamics of cavitation microbubbles one can ascertain dynamic changes in turgor pressure of the cell. In many ways the technique supersedes the labor-intensive, low-success-rate pressure probe, or micro-indentation methods that are often not compatible with in-situ studies (Zimmermann et al. 1992; Shabala and Lew 2002) (List S1). Here, we utilize the CBM to determine relative changes in epidermal cell turgor pressure of intact fine root cells during dehydration over a range of water potential thresholds commonly experienced by plants during water deficit. In combination, we determine thresholds for recovery or cell death in the cortex and stele of fine roots using a fluorescence cell viability assay to link shrinkage and turgor loss with cell mortality.

Using soybean (*Glycine max*), an economically important crop species with known vulnerability to drought (Harrison Day et al. 2026), we investigated the sequence of early-onset fine root physiological changes that occur during dehydration. We aimed to identify the turgor loss point of epidermal cells of fine roots and establish how changes in turgor are coordinated with volumetric shrinkage and tissue-specific mortality of fine roots during drying. We hypothesized that the early cortical shrinkage has consequences for cellular function across the root cell-layers that may have implications for water uptake and viability following dehydration and rehydration. From these thresholds of mortality and shrinkage, we ultimately investigated the potential for recovery and terminal damage at the fine root level during dehydration to understand the fine-scale stages of acute drought onset below ground.

## Results

**Figure 1:**
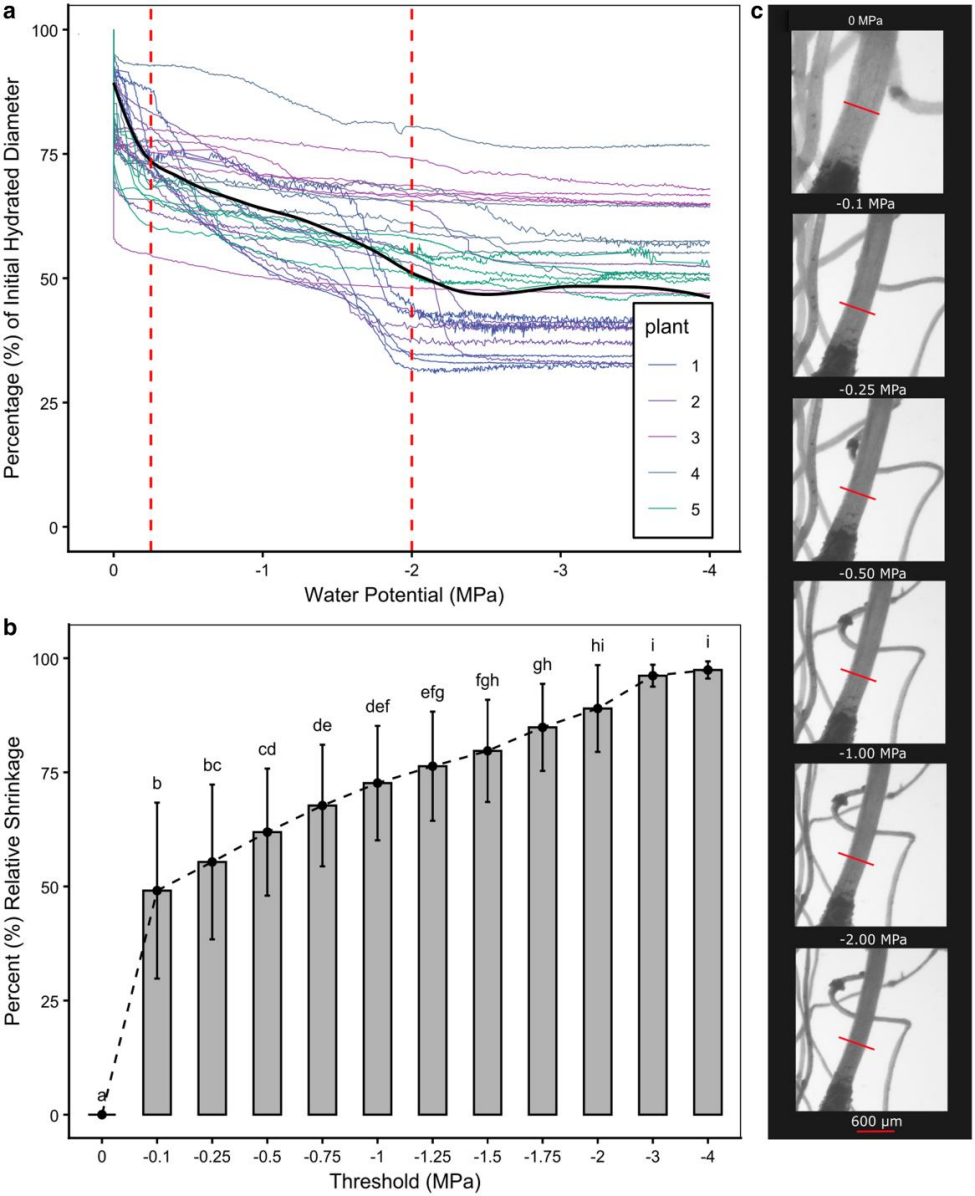
Fine root shrinkage in *G. max* over increasing xylem water stress. a) Total shrinkage of fine root diameters per individual root measured using optical dendrometry over declining water potential (Ψ MPa) from 100% diameter when fully hydrated, black line designating mean shrinkage (method = loess), vertical dashed lines indicating slope phase transition points. b) Mean fine root shrinkage ± SD (n = 28) as a percentage of relative shrinkage (normalized 0 to 100% from hydrated to final dry percentage per root), over key water potential Ψ thresholds: letters indicate ANOVA significant differences between means (*P* < 0.05). c) Representative images of fine root shrinkage from fully hydrated (top panel, −0.1 MPa to −2.0 MPa; red line indicates the initial hydrated root diameter.

Fine roots shrank by an average of 50.8% ± 12.37% of their total initial hydrated diameter (species values in Table S1) over the drought experiment where plants began at 0 MPa and ended at −4 MPa (Fig. 1a). We found 49.08% ± 19.27% SD of the relative shrinkage occurred by the time stem water potential had reached −0.10 MPa (Fig. 1b). By −0.25 MPa, the roots had completed 55.36% ± 16.95% SD relative shrinkage, 61.90% ± 13.91% SD by −0.5 MPa and 72.64% ± 12.54% SD relative shrinkage by −1.0 MPa. The highly nonlinear (GAM vs linear model ΔAIC = 31303.2) shrinkage process occurred in 3 distinct phases characterized by changes in the slope of mean shrinkage. The first phase was characterized by an initial steep decline in root diameter at stem water potentials (Ψ_stem_) of −0.10 MPa (Fig. 1c), followed by slower rate of shrinkage between −0.25 and −2 MPa, and a final shrinkage phase until −4 MPa (Fig. 1a).

**Figure 2:**
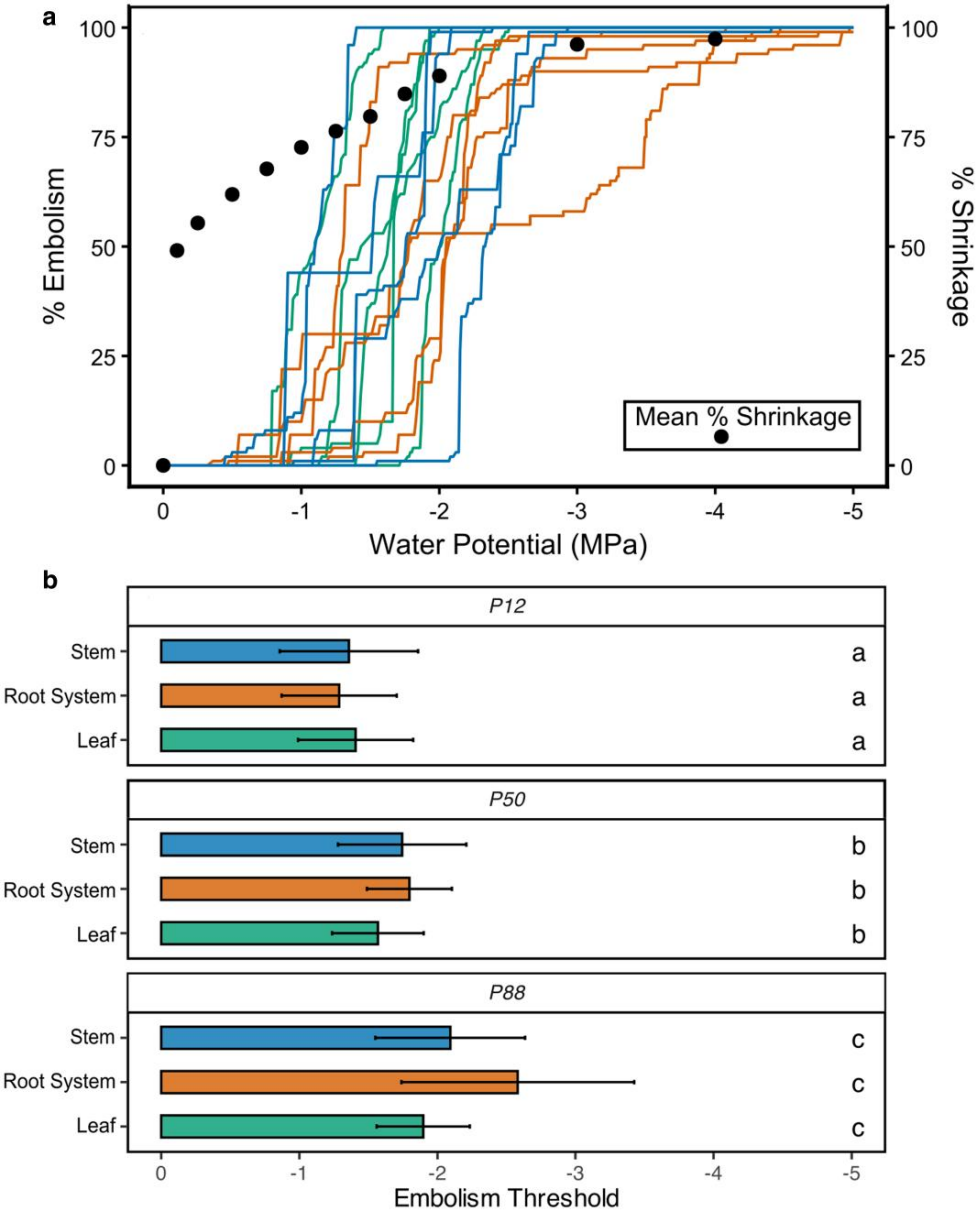
Xylem vulnerability of soybean (*G. max*) stems, roots, and leaves (n = 5 plants) in response to experimental drought conditions. a) Xylem vulnerability curves of stems, roots and leaves from intact plants (n = 5), aggregating each root system curve from the pixel weighted means of ∼10 fine roots; overlaid black points indicate mean root shrinkage over the same water potential scale. b) Mean *P*_12,_  *P*_50,_ and *P*_88_ ± SD for stem (blue), root system (orange), and leaf (green) xylem, calculated from the vulnerability curves, letters indicate ANOVA significant differences between means (*P* < 0.05).

We found no statistical differences in xylem vulnerability to embolism between leaves, stems, and roots (Fig. 2; *P* > 0.05 for each tissue type at *P*_12_*, P*_50_, and *P*_88_). Most root shrinkage occurred before any loss of conductivity resulting from xylem embolism (Fig. 2a), with >76% mean total root network shrinkage occurring by the point of incipient embolism (*P*_12root_ = −1.29 MPa; Table S2). Although root network means mirrored whole plant vulnerability to embolism, individual fine roots within the network showed broad variation in the timing of embolism onset (Fig. S2).

**Figure 3:**
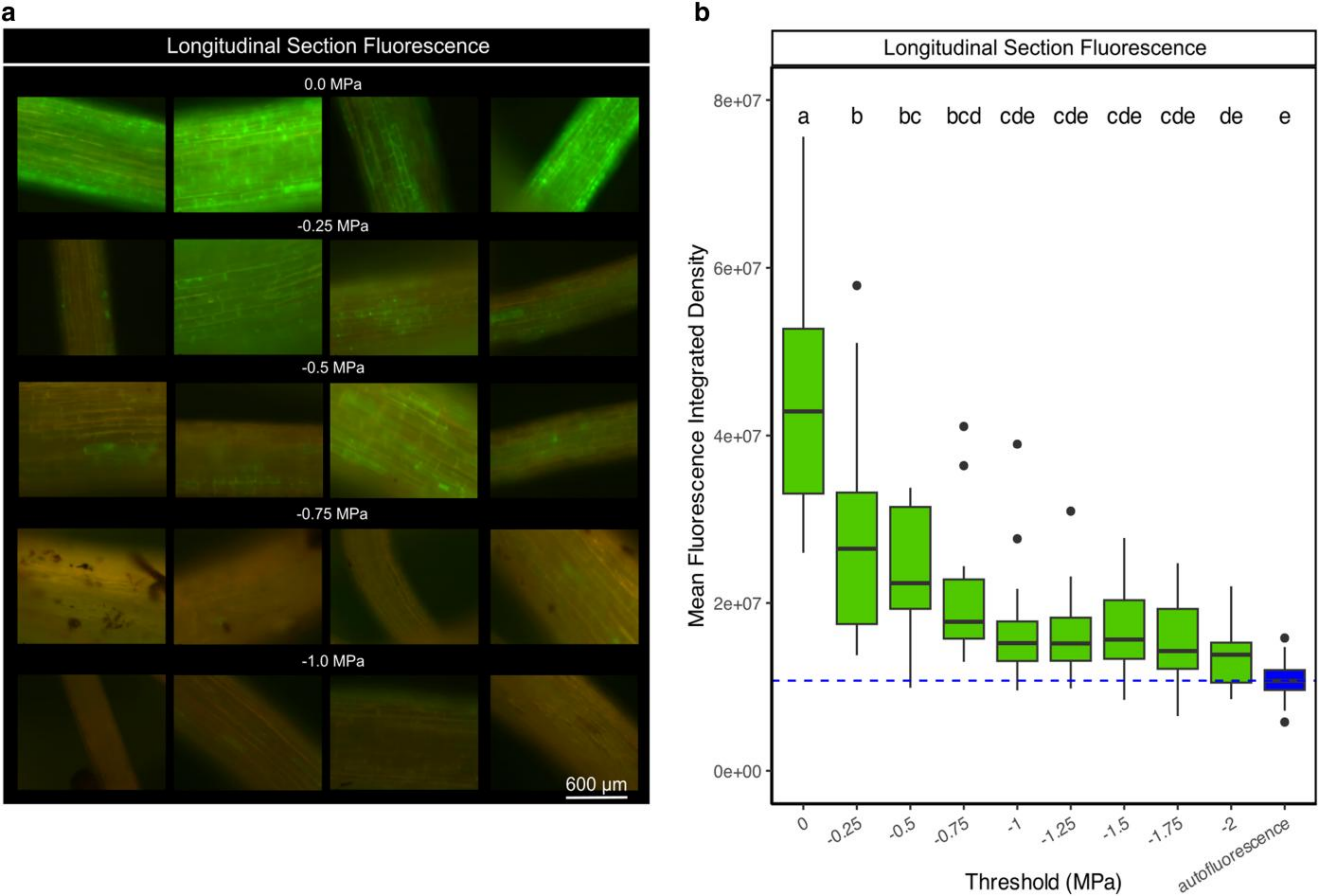
Fluorescein diacetate (FDA) viability staining of *G. max* fine roots in longitudinal section showing epidermal and cortical cell viability over declining Ψ_stem_. a) Representative FDA-staining of fine roots in longitudinal section (sampling a new root per image and per Ψ) at different points in the dehydration sequence from 0.0 MPa to −1.0 MPa. Bright green pixels indicate an uptake of FDA in living cells, which declined with declining water potential. Bar = 600 µm, applied to all images. b) Box and whisker plots of mean fluorescence measured as a numerical value of fluorescence integrated pixel density (mid-line designates median values, box designates interquartile range, points show outliers) compared against autofluorescence of unstained cells. Letters indicate significant ANOVA differences (*P* < 0.05) between means. Dashed blue line indicates the mean background autofluorescence signal acquired from the integrated pixel density values from dead fine root tissue.

We then used a cell viability staining method (fluorescein diacetate) (Ruzin 1999) to determine the dynamics of fine root cell mortality during the same drought conditions. Longitudinal sections allowed us to detect metabolic activity in the epidermal cells and outermost cortex cells. We found that cell viability in these 2 tissues declined once Ψ_stem_ reached −0.25 MPa. Once the water potential declined to −1.0 MPa, the FDA fluorescence signal was not significantly different from background autofluorescence signal in dead tissue indicating cell mortality, therefore occurring prior to the whole-plant *P*_12_ (Fig. 3).

**Figure 4:**
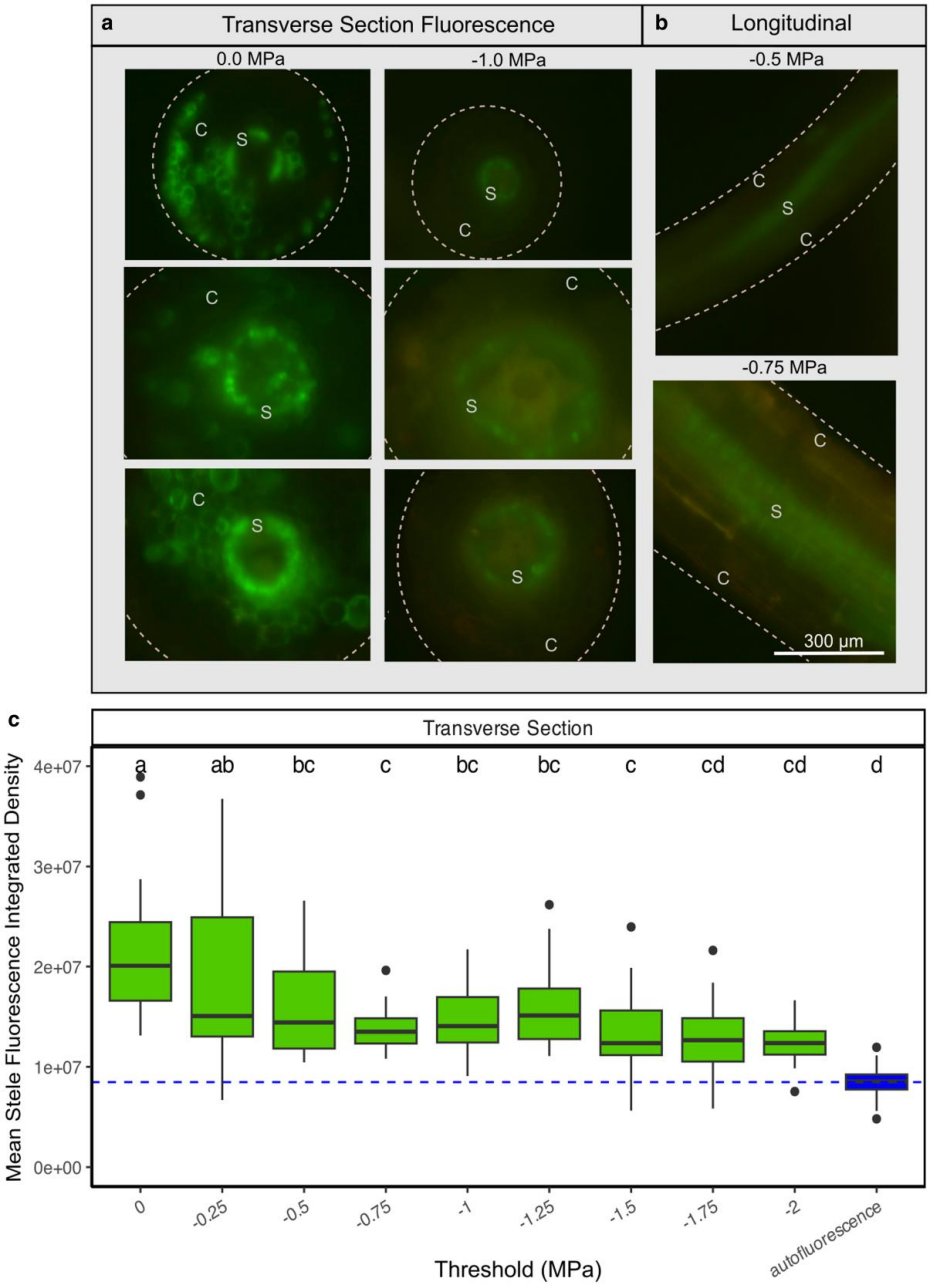
Fluorescein diacetate (FDA) stele cell viability staining of *G. max* fine roots when exposed to experimental drought conditions. a) Representative transverse sections showing the variability in FDA fluorescence (bright green) in fine roots at 0 MPa and −1.0 MPa. Dashed lines indicate the perimeter of the cortex (C = cortex, S = stele). (White 300 µm applies to all A and B microscopy images). b) Representative images of FDA fluorescence in longitudinal sections of roots at −0.5 MPa and −0.75 MPa. Fluorescence is primarily localized around and within the central stele. c) Box and whisker plots of mean FDA fluorescence signal measured as a numerical value of integrated pixel density (mid-line designates median values, box designates interquartile range, points show outliers) compared against autofluorescence of unstained cells. Letters indicate significant ANOVA differences (*P* < 0.05) between means. Dashed horizontal line designates mean autofluorescence integrated pixel density (dead cell value).

We also observed the FDA fluorescence in transverse section to compare cell viability between different tissue zones within fine roots, revealing divergent patterns of cell viability in stele cells within the endodermis (Fig. 4) compared with the cortex and epidermal cells (Fig. 3). Cortical and epidermal cells lost viability at less negative Ψ_stem_ values (determined by the water potential threshold where ANOVA showed significant overlap with the autofluorescence signal; ca. −1.0 MPa) compared with stele cells inside the endodermis, which lost function at −1.75 MPa, aligning with the whole-plant *P*_50_ (Fig. 4). Cortex and epidermal cell FDA fluorescence signal declined significantly (*P* = 0.4.12 × 10^−8^) from hydrated to −0.25 MPa (Fig. 3), whereas cells within the stele showed no significant loss of fluorescence over the same range of water stress (*P* = 0.77) (Fig. 4). Cortex cell mortality occurring prior to stele cells was most obvious in longitudinal sections of very fine roots (<400 μm) where there was less tissue to scatter and obscure the fluorescence signal (Fig. 4b) and where the surviving stele cell FDA fluorescence signal could be seen transmitted through the intact dead cortex tissues.

**Figure 5:**
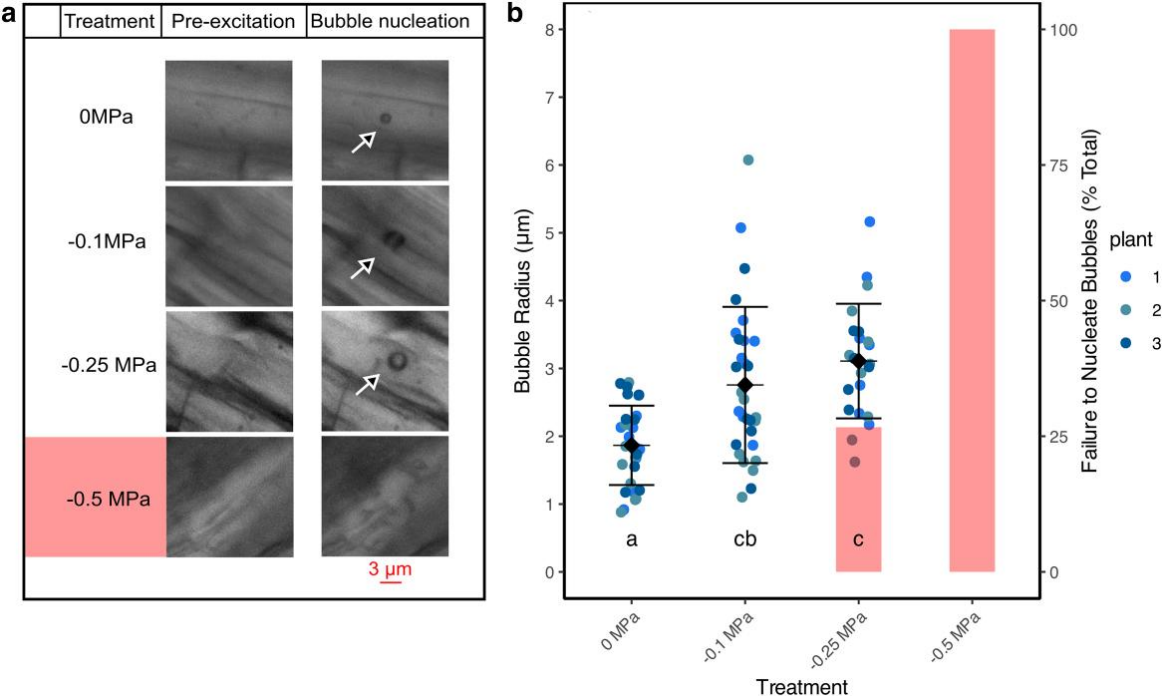
Changes in fine root cell turgor pressure in response to desiccation using cavitation bubble manometry, where microbubbles are nucleated within the cytoplasm of living cells. Microbubble growth is constrained by the pressure of the surrounding liquid, where lower turgor pressure results in larger microbubble radius. a) Representative transmitted light images before and after microbubble nucleation in fine root cells at different water potentials. Arrows indicate bubble location. Bar = 3 µm, applied to all images. At water potentials beyond −0.5 MPa spherical microbubbles would not form, indicating a complete loss of turgor pressure. b) Mean microbubble radius increased as fine root cells dehydrated, indicating a reduction in turgor (n = 3 plants; each point represents a microbubble nucleated in a different fine root cell). Opaque bars indicate the percentage failure rate to nucleate spherical microbubbles that rapidly dissolve into solution, lines designate means and SD. Letters indicate ANOVA significant differences (*P* < 0.05) between means.

We used cavitation bubble manometry (CBM) (Brodersen et al. 2025) to monitor changes in intact root epidermal cell turgor pressure when exposed to experimental drought and recovery and repeating the same conditions used in the FDA experiment. Microbubble radius increased significantly with declining water potential, which indicated a decline in cell turgor (Fig. 5). At a xylem water potential of 0 MPa, mean microbubble radius was 1.87 μm ± 0.58 μm SD, then increased to 2.76 μm ± 1.15 μm SD as soon as psychrometers registered a xylem water potential decline (−0.1 MPa), and increased again to 3.11 μm ± 0.85 μm by −0.25 MPa, all indicating a steep decline in turgor pressure in response to desiccation (Fig. 5). At −0.25 MPa we observed a 25% microbubble nucleation failure rate. Instead, the light pulse resulted in an aspherical, static void rather than a spherical bubble that rapidly dissolves in liquid-filled cells under turgor pressure. By −0.5 MPa, we were unable to successfully nucleate any spherical microbubbles, indicating a complete loss of turgor pressure and sufficient liquid water necessary to generate a microbubble.

**Figure 6:**
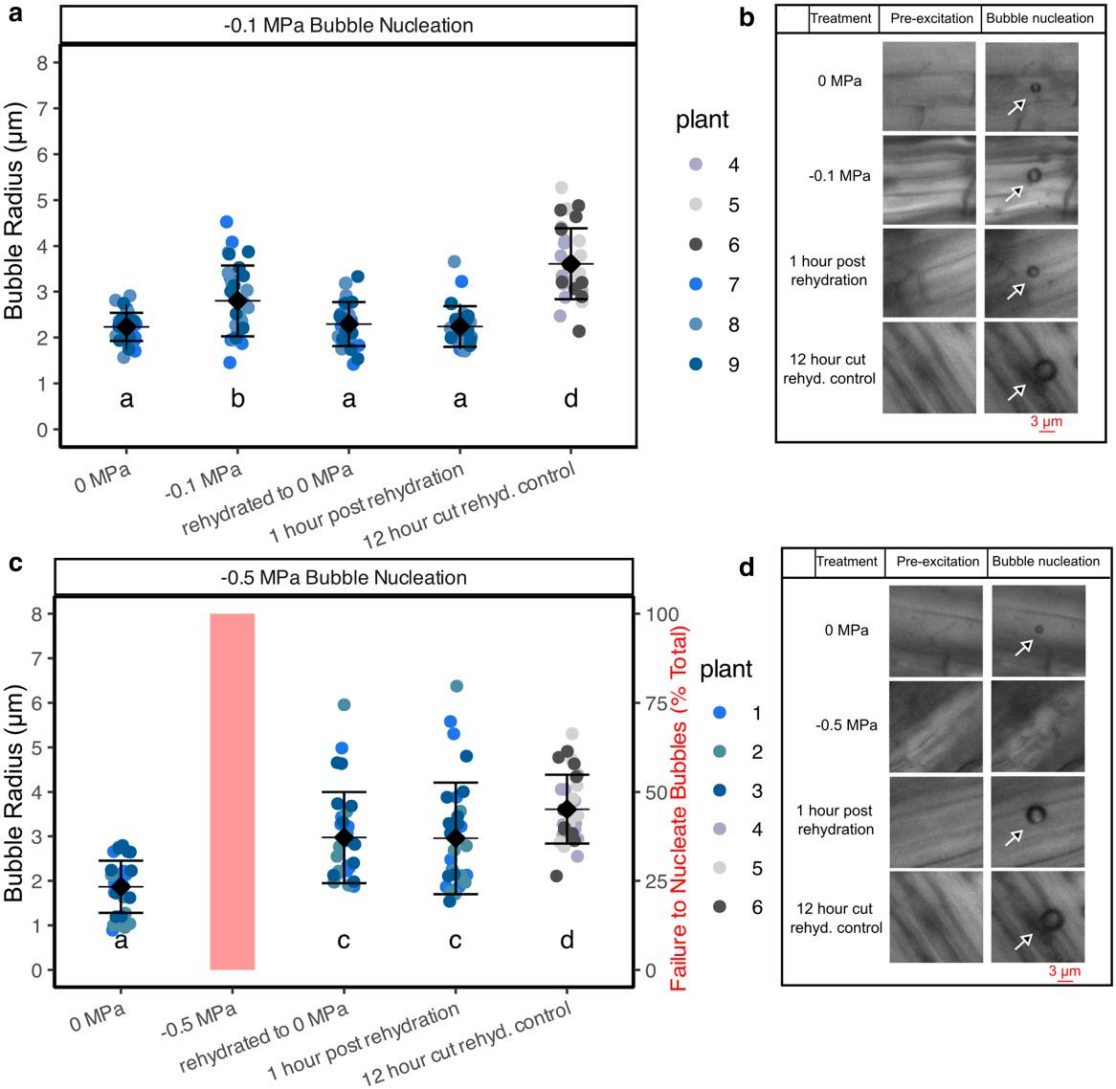
Loss of fine root cell turgor pressure in response to desiccation that exceeds a cell mortality threshold of −0.5 MPa, and subsequent failure of turgor pressure recovery after rehydration. Intact root cortical turgor loss and viability following dehydration and subsequent rehydration using the cavitation bubble manometry (CBM) technique and fluorescein diacetate (FDA) viability staining. a) Dynamics of fine root cell turgor pressure estimated from changing microbubble radius in plants dried to −0.1 MPa and rehydrated to 0 MPa. Microbubbles increase in response to dehydration and then decrease after rehydration. c) Microbubble radius in fine roots of plants dried to −0.5 MPa and rehydrated to 0 MPa. Microbubble radius, and therefore turgor, fails to recover in fine roots when plants exceed the −0.5 MPa threshold. Letters indicate significant ANOVA (*P* < 0.05) differences between means, lines designate means and SD, pale-opaque bars indicate percentage failure to nucleate bubbles per water potential. b, d) Representative images of fine root cells before and after microbubble nucleation at different water potentials; −0.1 MPa (b) and −0.5 MPa (D: continuation from experiment Fig. 5a, repeat images 0 and −0.5 MPa). Arrows indicate the microbubble nucleation location. Scale bar applies to all images, same representative 12 h image used in B and D.

We then set out to determine if epidermal cells could regain turgor following a cycle of mild desiccation and rehydration at thresholds that should not have resulted in significant losses in cell viability based on observations from our shrinkage and FDA staining experiments. Fine roots were dried to incipient xylem water potential decline (−0.1 MPa), which corresponds to when fine root cells begin to lose viability, primarily in the cortex (Figs. 3 and 4). Microbubble radius increased significantly (*P* = 0.001) when root water potential dropped to −0.1 MPa, indicating a decrease in turgor, and then returned to the same initial radius (*P* = 0.999) when rehydrated (Fig. 6a), suggesting a full recovery of cell turgor pressure was possible over this timescale and water potential threshold exposure (Fig. 6b). Roots were then dried to −0.5 MPa, at which point epidermal cells lost turgor, and enough water was also lost from the cell such that microbubbles would not form. When rehydrated from −0.5 to 0 MPa, we could generate microbubbles, but these were significantly larger (*P* = 0.015) than microbubbles that form in well hydrated roots (Fig. 6c), indicating an inability for the epidermal cells to fully regain turgor after being exposed to −0.5 MPa (Fig. 6d). We then excised fine roots from the plants and allowed them to dry in air for 12 h and then immersed them in water. Epidermal cells absorbed sufficient water such that microbubbles could form, indicating a capacity for dead cortical cells to regain some hydration state. However, these microbubbles were significantly larger (*P* < 0.0001) (3.61 μm ± 0.78 μm radius) than any observed in living cells, and similar in size to microbubbles nucleated in water. Thus, these epidermal cells were likely at or close to atmospheric pressure.

**Figure 7:**
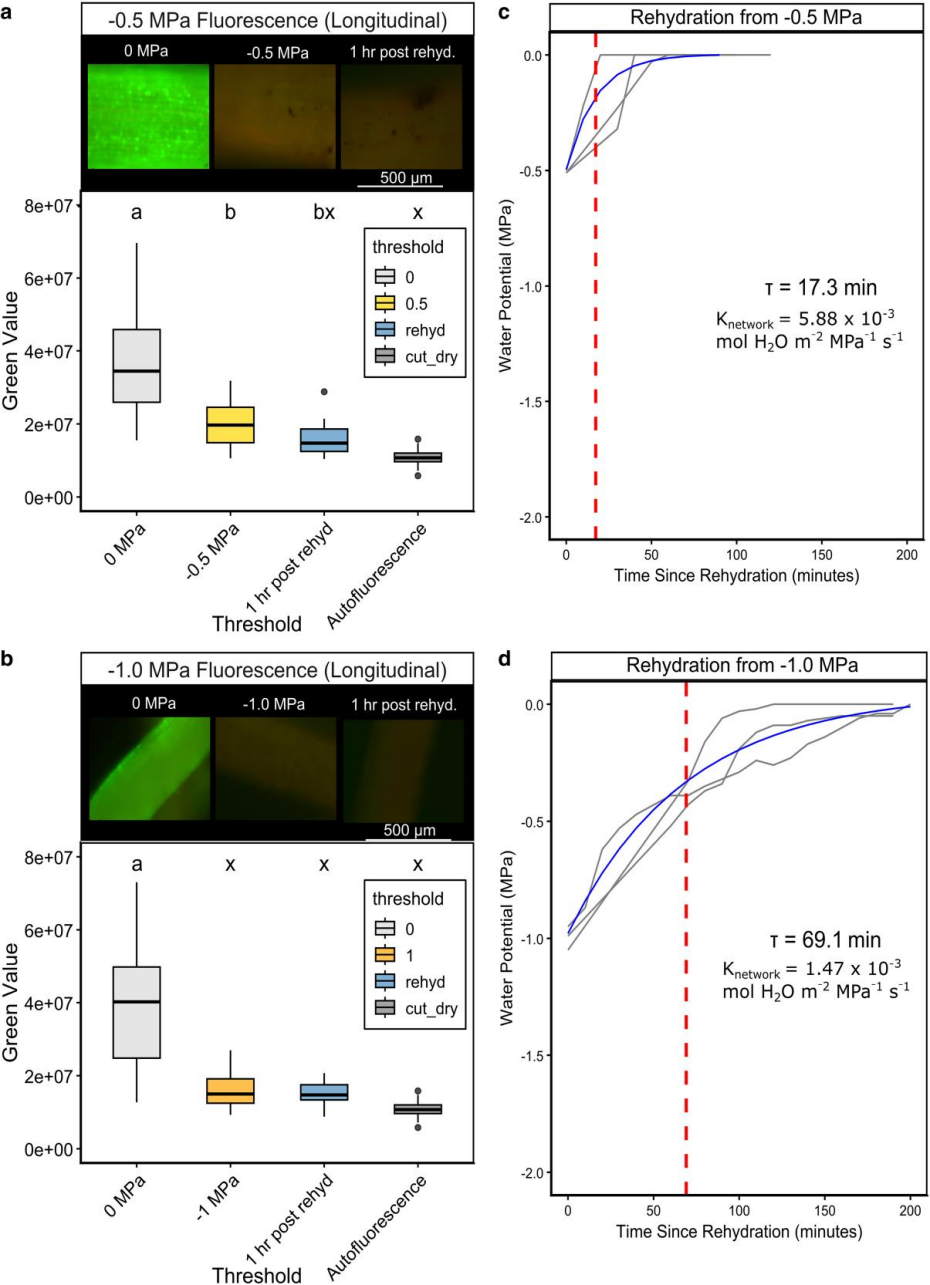
Loss of fluorescence-indicated cell viability during drying and rehydration, presented as box and whisker plots, revealing no recovery of cell viability. a, b) Fluorescence-indicated cell viability when fully hydrated, at −0.5 MPa (a) or −1.0 MPa (b) thresholds of water deficit and following rehydration, with representative fluorescein diacetate (FDA) stained longitudinal sections of intact roots per treatment (box plot center line = median; box limits = upper and lower quartiles; whiskers = 1.5 × interquartile range; points = outliers). Letters indicate ANOVA significant differences (*P* < 0.05) between means, bar = 500 µm, applied to all microscopy images. c, d) exponential rate of rehydration (plants = gray, mean = blue) of stem psychrometry from the imposed water stress following re-saturation of the root network. Red dashed line indicates τ mean time constant, text displays K_network_ hydraulic conductivity per threshold.

Dehydration treatments (examining epidermal and cortical cells in longitudinal section) from 0 to −0.5 MPa significantly reduced FDA fluorescence signal (*P* < 0.001) but did not result in complete loss of fluorescence. Fluorescence in these roots did not recover when rehydrated to 0 MPa, instead showing additional damage approaching mortality (Fig. 7a). Dehydration to −1 MPa resulted in complete fluorescence-indicated mortality of cortical and epidermal cells, which could not be recovered when rehydrated (Fig. 7b). Despite cortical cell damage in each treatment, stem water potential recovered exponentially, with a mean time constant (τ) of 17.3 minutes when rehydrated from −0.5 MPa and τ of 69.1 minutes when rehydrated from −1.0 MPa. We observed a root network capacitance of 6.10 mol H_2_O g^−1^ MPa^−1^ ± 1.67 SD; this capacitive rehydration was rapid and nonlinear (Fig. S3), mirroring the nonlinear optical dendrometry shrinkage observed during dehydration. This root network capacitance corresponded to a mean hydraulic conductance per root network mass (*K_network_*) of 5.88 × 10^−3^ mol H_2_O g^−1^ MPa^−1^ s^−1^ when rehydrated from −0.5 MPa (Fig. 7c). Plants showed a *K_network_* of 1.47 × 10^−3^ mol H_2_O g^−1^ MPa^−1^ s^−1^ when rehydrated from −1.0 MPa (Fig. 7d), representing a 75% reduction in hydraulic conductance between the −0.5 MPa and −1.0 MPa thresholds of cortical/epidermal cell mortality.

## Discussion

Here we document the physiological behavior of fine roots during the early stages of water deficit in soybeans. Fine root diameters shrank rapidly when exposed to air, completing over 50% of their relative shrinkage by −0.25 MPa, and 75% by −1.0 MPa Ψ_stem_ (Fig. 1). This water loss occurred mostly in epidermal and cortical fine root cells, which lost turgor and cell viability during this stage of drying (Figs. 4–7). By a Ψ_stem_ of −0.5 MPa, root epidermal cells lost turgor and exhibited significant cell mortality by −1.0 MPa. This mortality, however, was localized only to the epidermis and cortex, while cells within the stele remained viable until −1.75 MPa, closely corresponding with the formation of embolism in the xylem (whole plant *P*_50_). This rapid rate of change was likely influenced by soil removal, accelerating the cell processes that occur in soil during dehydration; with examining root-cell-soil interactions presenting a logical next step in understanding this process.

Despite the early shrinkage of cortical cells, stem water potential was fully recoverable from water potentials known to cause cortical mortality and epidermal turgor loss, indicating that damage to these cell layers did not preclude water uptake to the living stele cells and functional xylem. Fine root shrinkage apparently allows for the epidermis to retract from the surrounding soil matrix, physically decoupling fine roots from the surrounding soil, decoupling roots from soil matric potentials via capillarity, and theoretically slowing further exposure of the plant to declining water potentials. The survival of cells within the stele, combined with the ability for the cortex to retain a water absorption role even after death provides this species with the potential for rehydration and initiation of new conductive growing root segments provided rehydration occurs prior to terminal embolism in the xylem that coincides with cell death in the stele. Furthermore, death of large populations of cortex cells would reduce the respiration costs of fine roots (Desrochers et al. 2002) during water stress. This selective cell mortality is supported by previous observations in maize which documented survival of stele cells beyond cortex mortality thresholds (Facette et al. 1999), as well as selective cortex mortality along the length of maize roots during drying (Stasovski and Peterson 1991). The mass collapse of cortex at such minimal water stress suggests that these thin cortical cells operate with a “hydrostatic” skeleton to maintain shape, losing structure as soon as water potential falls below the turgor loss point, as shown in flower petals (Roddy et al. 2023).

### Plant survival given very early cortical damage

The rapid shrinkage and mortality of cortical cells in fine roots presents significant questions regarding how a plant can functionally manage cortex damage at such minimal water deficit. This observation poses mechanistic possibilities. First, that this cortical shrinkage and deformation occur very frequently, given that soybeans routinely experience water potentials within this range during transpiration (Wijewardana et al. 2019). Common mortality of parts of the root network may represent a contributing factor to the documented 30% to 50% annual global net primary productivity allocated to the turnover of fine roots (Gill and Jackson 2000). Given the high frequency at which fine roots experience water potential that would lead to cortex cell mortality, it is not surprising that allocation of resources to this root cohort for growth and absorption would be high.

An additional compounding possibility is that the endodermis presents a significant hydraulic resistor within the roots (Schreiber et al. 1999), so that some parts of the external cortical and epidermal cell layers are not in perfect equilibrium with the xylem water potential. Instead, the soil water potential and external environment could be exerting stress on these cell layers prior to the xylem water potential decline, with the early shrinkage here particularly amplified in artificially dry laboratory vapor pressure conditions. Here we could attribute the spatial localization of drought-induced cell mortality to the ability of the Casparian strip to more tightly regulate the loss of water, thereby protecting living cells within the stele from excessive declines in water potential (Schreiber and Franke 2011). The survival of stele cells until *P*_50_ aligns with the correlation of xylem embolism and cell mortality in leaves (Brodribb et al. 2021), while the early cortical mortality represents a clear divergence from this pattern. This suggests that mortality in tissues with tighter hydraulic control (stele root cells protected by the Casparian strip and leaf parenchyma protected by cuticle and stomata) is closely linked with xylem water potential. The extremely early cortex mortality, likely accelerated by bare-root laboratory conditions, supports the hypothesis that dynamic changes in epidermal and cortical cell layers are driven by the external environment rather than internal xylem hydraulic status. In this case, roots could utilize localized saturation and maintain cortical viability where water persists, and shrinkage would initiate as soon as soil water potentials begin to locally decline. This is supported by observations using MicroCT (Duddek et al. 2022) that document root hair shrinkage at minimal soil matric potentials between −0.10 and −0.31 MPa, although larger roots did not shrink until significantly greater soil water stress (−1 MPa) highlighting shrinkage variation across root orders. Localized cortical shrinkage and cell death could function to decouple plants from the hydraulic stress exerted on the plant by heterogeneously drying soil, while maintaining uptake in saturated regions; allowing uptake zones for the plant to dynamically move as the soil dries.

Although it is difficult to generalize from a vulnerable annual herbaceous crop species, Harrison Day et al. (2026) showed similar shrinkage patterns across diverse species spanning lycophyte to woody angiosperms; however, this study similarly utilized bare roots and may also reflect potentially accelerated shrinkage in laboratory drying conditions. In a porous substrate, rapid fine root shrinkage would allow roots to efficiently buffer xylem from soil water potentials as tension in the soil increases; however, this pattern may be counterintuitive in clay soils with slower saturation decline and a greater predisposition to waterlogging. Barrientos-Sanhueza et al. (2024) highlight lowered fine root turgor loss point and capacitance as traits determining drought tolerance in hydroponically grown grapevine. The intersection of root turgor, soil media, and soil matric potentials presents key avenues for research surrounding shrinkage in diverse root types and substrates. The very rapid shrinkage here suggests that K_soil-root_ decreases as soon as the water potential begins to decline; this diverges from Bourbia et al. (2021), who instead identify a significant tipping point below −1 MPa, again reinforcing that soil removal here may be impacting rate of change.

### Early cortical mortality not fatal for the whole root network

Functionally, early cortical cell death may not be unexpected or catastrophic for the long-term viability of the plant. Previous research has described cortical senescence as a routine component of root development (Bingham 2007). Roots often show a developmental transition from absorptive to transportive, structural roots during secondary and tertiary growth, losing their cortex and developing peridermal cell layers (Guo et al. 2008; Harrison Day et al. 2026). In this case, the selective preservation of cell viability inside the stele would prolong the delivery of water, nutrients, and carbon necessary for the survival of meristematic regions within the root pericycle, and lateral absorptive roots may theoretically be replaced when soil hydraulic conditions become favorable (De Smet 2012). Here, the early death of cortex may present a key step in the cortical senescence transition of roots from absorptive to transportive function (Stasovski and Peterson 1991). The living stele cell layers may then continue the transition to secondary peridermal growth. Jones et al. (2025) highlight particularly in Poaceae that the root cortex is a “dispensable” tissue. Thus, the cortical layers of fine roots likely represent an ultimately transient cell layer during the lifespan of the growing root network, protecting the essential meristematic regions within the root during water deficit.

Importantly, we show here that damaged epidermal and cortical cells still retained properties to regain a hydrated state and could then allow water to be redirected through them to the surviving stele, with water moving across the epidermis and cortex cells and through void spaces theoretically left during lacunae formation (Cuneo et al. 2016), allowing the complete recovery of Ψ_stem_. However, dehydration between −0.5 (epidermal turgor loss with reduced epidermal and cortical fluorescence) and −1.0 MPa (epidermis and cortex mortality but stele viability) reduced the rate of hydraulic conductance (K_network_) by 75%. This suggests that the gradual death of the external cell layers, and the barrier properties of endodermal suberin layers, slow but do not entirely prevent rehydration (Schreiber et al. 2005). The highly variable onset of embolism across the network may also contribute to this reduced K_network_ given that some root segments became embolized before −1 MPa, before the *P*_12_ of −1.29 MPa. Our rehydration kinetic measurements of capacitance (6.10 mol H_2_O m^−2^ MPa^−1^ ± 1.67 SD) reinforce that damaged cortical cells maintain some potential for capacitive water storage, with fine root collapse potentially providing a large part of this volume. Our soybean root network capacitance falls within the range of the 2 published root network capacitance values reported by McCarthy et al. (2025) in the conifers *C. rhomboidea* (17.2 mol m^−2^ MPa^−1^) and *P. radiata* (3.4 mol m^−2^ MPa^−1^). These conifer species are known to also exhibit early fine root shrinkage patterns (Harrison Day et al. 2026) similar to those seen here in soybean, suggesting there may be a common link between fine root shrinkage and root capacitance across species. However, the slowed rehydration over increasing drought suggests that new fine root segments must be regrown from surviving meristems to fully recover hydraulic conductance.

## Conclusions

These fine root findings contribute to a broadening view of dynamic root behavior that highlights physiological responses beginning early during water deficit, and well before they appear in leaf and stem tissues. Significant work has gone toward investigating root traits across diverse scales; spanning cell proliferation and programmed death to landscape scale turnover and carbon sequestration (Drew et al. 2000; Gill and Jackson 2000; Freschet et al. 2021). Despite this scope, and the essential nature of root behavior in predicting plant survival in uncertain environments, a mechanistic and functional physiological link has been missing bridging the cell-level and macro scales. Here we provide a conceptual steppingstone, investigating the relationships between cell turgor and viability in combination with whole-plant measurements of vulnerability to embolism and water potential to show that individual fine roots cell layers are playing a highly specialized and dynamic role in plant hydraulics during the onset of drought.

## Materials and methods

### Plant material

Soybean (*Glycine max* (L.) Merr., var. “Sloan”) seeds were obtained from the USDA Germplasm Repository (Strain: PI 548,616, Maturity Group: II) and grown from seed at the Yale School of the Environment Greenhouse facilities until 4∼7 weeks old in 14-hour daylength and watered daily. Plants were grown in in 3L pots in peat-based medium containing perlite and vermiculite (Premier Pro-Mix HP; Premier Horticulture Inc., Quakertown, PA, US) with a slow-release (Scotts Miracle-Gro, Osmocote). Experiments were conducted on intact fine roots < 2 mm diameter, encompassing 95+ % of the root network for all plants. All measurements were conducted on regions of root 2 + cm away from the growing root tip to ensure functional xylem and fully expanded epidermal and cortical cells, and 5 + cm below the root collar to avoid secondary roots without cortex, to ensure focus on absorptive regions of root. Experiments were conducted on bare roots to allow access to fine root material. Roots were exposed to a uniform laboratory relative humidity of ∼50% at 18 °C ± 3 °C under ∼30 µmol m^−2^ s^−1^ PPFD, with the understanding that artificially dry air may accelerate epidermal and cortical shrinkage processes that normally occur during soil drying.

Five plants were used to determine the vulnerability of the xylem network using the Optical Vulnerability (OV) method (Brodribb et al. 2016); 5 plants of the same developmental age were used to determine root shrinkage using the Optical Dendrometry (OD) method (Bourbia and Brodribb 2023); 3 plants were used to collect whole plant simultaneous water potential Ψ data; 3 plants were used to assess root capacitance; roots from 15 plants were used to determine cell viability using fluorescein diacetate (FDA) staining when dried to specific water potential thresholds, and in some cases rehydrated, and 9 plants were used with the CBM.

### Measurement of stem water potential

For all dehydration experiments, water potential Ψ was measured using stem psychrometry (PSY1, ICT International, NSW, Australia). A region of epidermis at the base of the stem close to the root collar, before the first mature set of leaves, was removed with a razor blade to expose xylem and was washed with distilled water. The psychrometer chamber was clamped onto the stem and sealed with layers of parafilm. To ensure an airtight seal and to prevent crushing of the stem, baffles of rolled parafilm were placed on either side of the stem over the sealed parafilm, and then a foam gasket (2 cm × 2 cm × 5 mm) placed over the top before clamping. For OV, OD, CBM, and FDA experiments, stem psychrometer values were applied to roots under the assumption that water potential gradients equilibrate within the plant following stomatal closure.

### Assessment of whole plant water potential equilibrium

**Figure 8:**
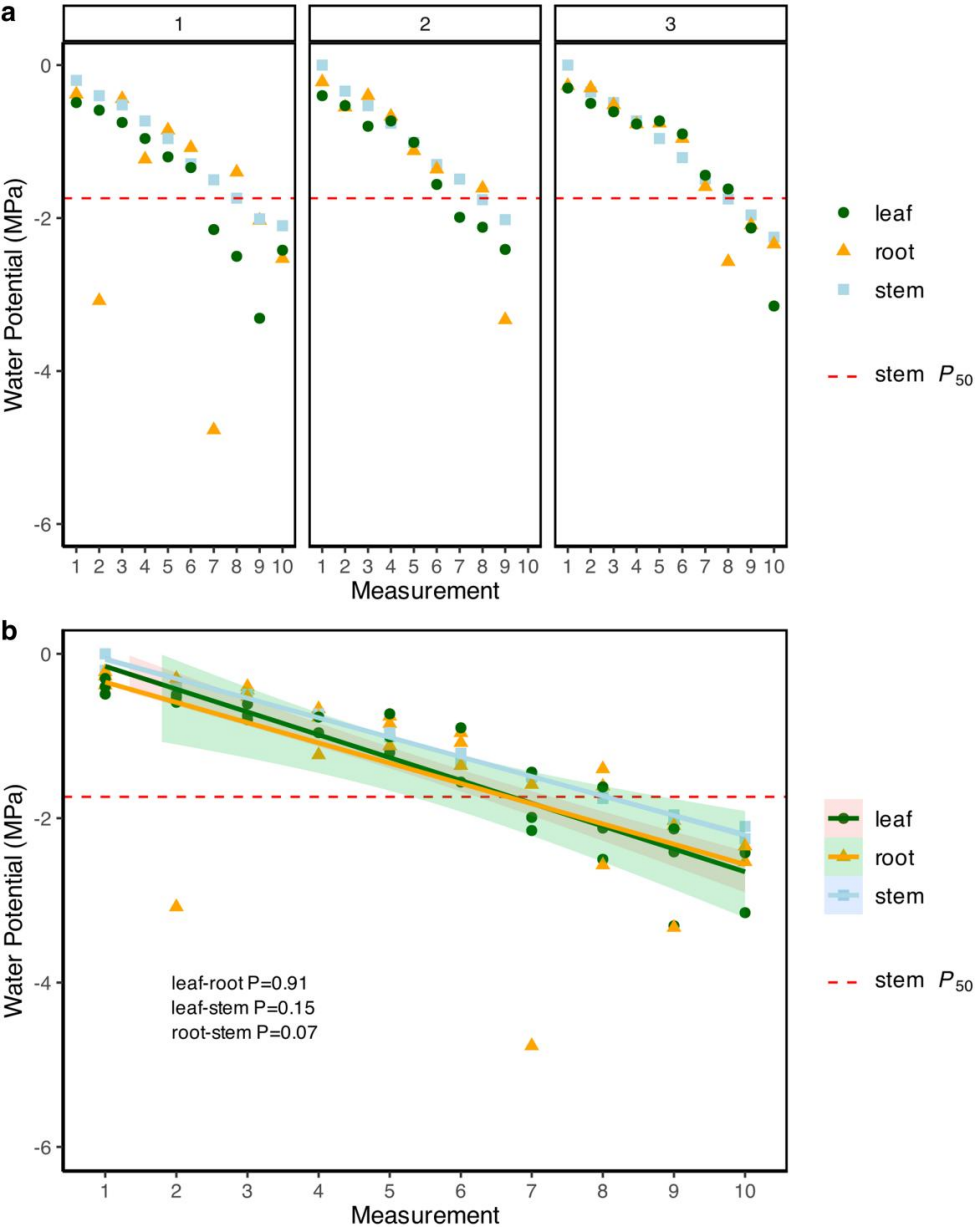
Simultaneous water potential measurements of leaves, roots, and stems using psychrometry in 3 intact soybean (*G. max*) plants to capture each organ at the same thresholds of water stress, showing consistent whole-plant water potential rates of drying. a) Rates of drying in roots, stems, and leaves for the 3 different plants (1 to 3). b) Changes in water potential of leaves, roots and stems during the dehydration experiment, regressions show means ± SE shading (n = 3 plants per water potential measurement), indicating no significant differences between pairwise (ANCOVA-adjusted *t* tests) comparisons of mean slopes.

To ensure that laboratory-exposed fine roots did not deviate from stem xylem water potentials given their large evaporative surface area, we simultaneously measured water potential of stems, leaves and fine root during drying. Three plants were dried on the laboratory until the water potential reached −2 MPa, a threshold known to induce ca. 88% of the stem xylem embolism (∼*P*_88stem_). Leaf and root samples were collected at −0.25 MPa intervals determined by the ICT stem psychrometer per plant, starting at 0 MPa immediately following soil removal. Root samples (five 3-mm sections per measurement) and leaf disks (from fully expanded leaves) were placed inside point psychrometer chambers (JRD Merrill Specialty Equipment, Logan, UT, USA). Tissue samples were allowed to equilibrate inside the chamber until readings stabilized, typically after 3 to 4 h. Complete dry-down experiments from 0 MPa to −2 MPa took ∼12 to 16 h per plant. Within-plant rates of water potential decline of leaves, stems, and fine roots were highly consistent (*P* > 0.05) until Ψ_stem_ exceeded −1.75 MPa, which coincided with the measured *P*_50_ and the presence of significant xylem embolism (Fig. 8), (Fig. S1). Ψ_leaf_ and Ψ_root_ dropped below Ψ_stem_ only after reaching the whole-plant *P*_50_. Individual root segments from optical vulnerability data were examined to find total variance in vulnerability to embolism, and some root segments were found to reach *P*_50_ as early as −0.55 MPa (Fig. S2). The outlying 2 root examples in the dry-down (Fig.8, Plant 1) dataset likely represent roots that embolized early, becoming isolated from the plant (Fig. 8a), while the broader root network with intact xylem connection remains at the whole-plant water potential. Importantly, early and variable shrinkage could not be attributed to differential xylem tensions across the network.

### Optical vulnerability method

The Optical Vulnerability (Brodribb et al. 2016) method was used to measure the vulnerability to embolism of roots, leaves, and stems of intact plants (n = 5). Four OV cameras were used per plant: 1 camera on a fully expanded leaf, 1 camera mounted to the main stem directly adjacent to the second mature leaf node, and 2 cameras capturing ∼5 fine roots per field of view (10 total). For stems, epidermis (8 × 2 mm) was removed to expose xylem, protected with ultrasound gel (Tensive Adhesive Gel Fairfield, NJ, USA) to prevent dehydration and improve light transmission, and was clamped between glass slides. OV images were captured in five-minute intervals using reflected light mode until the cessation of embolism events and failure of psychrometers at low water potentials. A mature leaf was clamped into an OV camera and imaged using transmitted light. Intact, washed fine roots were placed between glass slides buffered with gel and imaged in OV cameras using transmitted light. Vulnerability curves were reconstructed for each organ (Brodribb et al. 2017; Harrison Day et al. 2024) with the Open Source Optical Vulnerability plugin (Lucani 2025) for ImageJ (Schneider et al. 2012). A mean percentage embolism from ∼10 roots per plant was aggregated by pixel weighting to account for larger percentage flow in larger roots. The total area of pixel changes at the cessation of drying was considered as the point of complete embolism, resulting in 100% loss of conductance. The Ψ at which 12%, 50%, and 88% of the tissue had embolized (*P*_12_, *P*_50_, *P*_88_) were extracted for organ comparison, using Ψ_stem_ captured using psychrometry.

### Fluorescence cell viability staining

Fluorescein diacetate (FDA) (Sigma-Aldrich, Schnelldorf, Germany) was used as a rapid stain to assay the viability of cells through fluorescence-based visualization of cellular activity during drying. FDA is incorporated into viable cells where it is hydrolyzed by esterases to produce fluorescein, fluorescing the cytoplasm green under blue-light (Ruzin 1999). A stock solution of FDA was made by preparing 10 mg of dry-mass FDA in 10 mL acetone to give a 1 mg/mL stock solution, which was stored at 4 °C. A working solution was made by diluting 100 μL FDA stock in 100 mL deionized water to a final concentration of 1 μg/mL FDA. A fresh working solution was made for each plant to avoid FDA degradation in water. Six roots were sampled for per plant per water potential treatment (below). For each plant, root segments were excised from the root system with a razor blade. Transverse sections and longitudinal sections were prepared per root to assess differential cell viability. Freehand transverse cross sections (∼30- to 50-μm thickness) were immersed in FDA solution for 15 min and were immediately used to study cell viability in the stele. Longitudinal segments of the same roots ∼2 cm in length were stained with FDA solution for 15 min for observing epidermis and cortex viability. Sections were rinsed in DI water and mounted on a glass slide and imaged using blue light (cube U-MNIB, excitation filter 470 to 490 nm, dichroic mirror 505 nm, barrier filter 515 nm) using a fluorescence microscope (Olympus BX60). Images were collected with a shutter speed of 1″, ISO 1,250, and 20× magnification. Images were analyzed in ImageJ, extracting only the green RGB channel. A 1,000 × 1,000-pixel region of interest (ROI) was set to avoid root size-related saturation effects and placed over the stele in transverse sections or the cortex in longitudinal sections. Fluorescence intensity was calculated as the integrated density within each ROI.

Fluorescence experiments were conducted to measure complete dry-down, and recover from either −0.5 or −1.0 MPa (3 × plants per experiment). For the dry-down experiment, fluorescence was measured every 0.25 MPa from fully hydrated (0 MPa) to −2 MPa, with whole plants taking 12 to 16 h to dry. For the recovery experiments, fluorescence was measured at 0 MPa, again at the treatment threshold (−0.5 or −1.0 MPa) and finally when rehydrated. Following the dry treatments, roots of each intact plant were immersed in water and leaves covered with damp paper towel, recording the time taken to rehydrate. Once the stem psychrometer had returned to 0 MPa, plants were allowed to equilibrate for an hour with roots remaining in water and then final root fluorescence measurements were taken. Additionally, control plants with no fluorescent stain were assessed for background auto-fluorescence at 0 MPa (3 × plants) and fully dry (3 × roots cut and airdried for 12 h) capturing 10 roots per plant.

### Root system capacitance

As per McCarthy et al. (2025), roots were washed and gently dried with paper towels and then placed in a plastic bag so that water potential decline was driven only by transpiration from stem and leaf tissue rather than evaporation from roots. Plants were slowly dried to approximately −0.7 MPa, a xylem tension threshold prior to significant root embolism, then were covered with black plastic bags and allowed to equilibrate for ∼ 2 h. The stem of the plant was then excised under de-gassed, deionized water 2 cm from the root collar, and then recut at a 45˚ angle, all while leaving the root system in the plastic bag to prevent rewetting of the roots. The cut stem was attached to water-filled tubing, allowing roots to draw water from a beaker on a computer-interfaced balance (Sartorius Practum 224 to 1S, ±0.0001 g) weighing every 5 s. After 5 h, a final Ψ_root_ measurement was taken by placing root into 8 Merrill psychrometer chambers (5 × 3 mm segments per chamber). A final root network dry mass was calculated after drying roots for 3 days in a 60 °C oven. Capacitance was calculated as


(1) $$C=\frac{m_{f}-m_{i}}{\Delta \Psi }$$

where *C* is capacitance expressed as mmol g^−2^ MPa^−1^, $\;\;m_{f}$ is final mass of water after rehydration and $m_{i}$ is initial mass before rehydrating, normalized to root network dry mass (g), ΔΨ is the change in water potential pre and post rehydration. Root network hydraulic conductance (*K*_network_) at −0.5 MPa and −1.0 MPa was calculated as


(2) $$K=\frac{C}{\tau }$$

where *C* is root network capacitance and τ is the time constant of the exponential rehydration curve from the fluorescence experiments (below), expressed as mol H_2_O g^−1^ MPa^−1^ s^−1^.

### Cavitation bubble manometry

We measured changes in turgor pressure of intact fine root epidermal cells over a dehydration gradient using Cavitation Bubble Manometry (CBM) Brodersen et al. (2025). A 1.3 µJ pulse of blue (460 nm) light from a modified MicroPoint laser (Andor, USA) was aimed at root cells on a custom-built microscope (SLMPlan, Olympus, Tokyo, Japan) (20× long-working-distance). The light pulse vaporized a small volume of cytosol, forming a vapor microbubble. According to cavitation bubble dynamics theory (Epstein and Plesset 1950; Li et al. 2011; Brodersen et al. 2025), maximum radius is constrained by the fluid pressure of the surrounding liquid, so that increasing radius corresponds to a decrease in cell turgor pressure. Microbubble radius and dissolution time was measured with a high-speed imaging camera (1,000 frames per second), capturing 1 second of data, (#acA640-750 um, Basler, Inc., Exton, PA). Bubbles were deemed within cells rather than water because they remained confined within cell boundaries (unlike freely moving water-only bubbles), and bubbles were difficult to nucleate in water alone.

The image sequence was then imported into ImageJ processing software (Schneider et al. 2012) and the first image where the microbubble was clearly visible was identified. An ellipse was then fitted to the bubble, measuring (Feret's diameter/2) to find the bubble radius, and these radii data were then compared between different treatments. Where multiple bubbles were nucleated in 1 cell, the largest bubble was measured. While we were not able to determine absolute values of turgor pressure, changes in microbubble radius have been determined as an accurate proxy with a rigorous grounding in cavitation bubble dynamics theory (Brodersen et al. 2025). Given that the relationship between microbubble radius and pressure is non-linear (Li et al. 2011), a small change in radius can be interpreted as a large change in absolute turgor pressure.

For CBM experiments, the root bundle was placed in a shallow petri dish filled with water, and intact roots were stained for 10 minutes in Neutral Red aqueous stain (∼0.005% w/v) (Mississauga, ON, USA) to increase the success of laser microbubble nucleation by increasing light absorption in the highly translucent roots. Neutral red was chosen as a low-toxicity stain with efficacy in root cells (Dubrovsky et al. 2006). After 10 minutes the remaining stain was washed off the roots. For each plant, measurements were made at 0 MPa with roots in water, at 0 MPa in air and as the plant dried to −0.1, −0.25, and −0.5 MPa. The root bundle was rehydrated (leaves covered with damp towels), and microbubbles recorded at 0 MPa and 1 h later. 10 microbubbles were measured per plant, per water potential threshold. We then repeated the protocol above in plants dried to only −0.1 MPa (recoverable fluorescence threshold). To provide a reference, we also tested microbubble formation in rehydrated, non-viable cut roots killed by air-dry overnight for >12 h (confirmed with FDA fluorescence) and then rehydrated those samples for 1 h in distilled water. We then nucleated and recorded microbubbles in a total of 30 rehydrated root epidermal cells.

### Statistical tests

The differences in P_50_ between organs were compared using a 1-way ANOVA of equal variance, considering organs statistically different when (*P* < 0.05). To compare the homogeneity of water potential across organs during the whole-plant dry-down, we performed an analysis of covariance (ANCOVA) using a linear model (lm). Pairwise comparisons of estimated marginal means were conducted to assess differences between organs throughout the dry-down, considering organs statistically different when (*P* < 0.05). Shrinkage of each individual root was visualized by taking of the maximum hydrated diameter in pixels as 100% (of hydrated diameter). A normalized scale was calculated by taking the final diameter at the sensation of drying and plotting each root on a scale of 0% shrinkage (diameter at fully hydrated) to 100% shrinkage (diameter at the cessation of drying). Thresholds were extracted at the initiation of xylem water potential decline (extracted as −0.1 MPa), −0.25, −0.5, −0.75, −1, −1.25, −1.5, −1.75 and −2 MPa. Statistical differences between means were calculated using ANOVA. The shape of the mean shrinkage was compared between a linear model and gam, taking the lowest AIC value as the better fit. Fluorescence was compared across groups using ANOVA, first by examining differences in mean fluorescence at 0 MPa, −0.25, −0.5, −0.75, −1, −1.25, −1.5, −1.75 and −2 MPa, and then by comparing fluorescence in the rehydrated plants at 0 MPa, −0.5 or −1 (per treatment) and in rehydrated roots. Bubble size was compared using ANOVA to examine differences at each treatment threshold. All statistical tests and data visualization were conducted using R (4.1.1) (R Core Team 2024).

## Supplementary Material

kiag132_Supplementary_Data

## Data Availability

The data that supports the findings of this study are available in the Supporting Information of this article.
