# Supplementary material for: Cortex death precedes stele failure in soybean fine roots during drought but does not prevent plant rehydration
Source: Plant Physiol. 2026 Mar 13;201(1):kiag132. doi: 10.1093/plphys/kiag132 (PMC13181395; doi:10.1093/plphys/kiag132)
Supplement: kiag132_Supplementary_Data [file kiag132_supplementary_data.pdf]

Supplementary data:

Plant Physiology Paper Title: Cortex death precedes stele failure in soybean fine roots during drought but does not prevent plant rehydration.

Authors: Beatrice L. Harrison Day, Christopher McCarthy, Timothy J. Brodribb, Madeline Carins-Murphy, Craig R. Brodersen

Supplementary Figures:

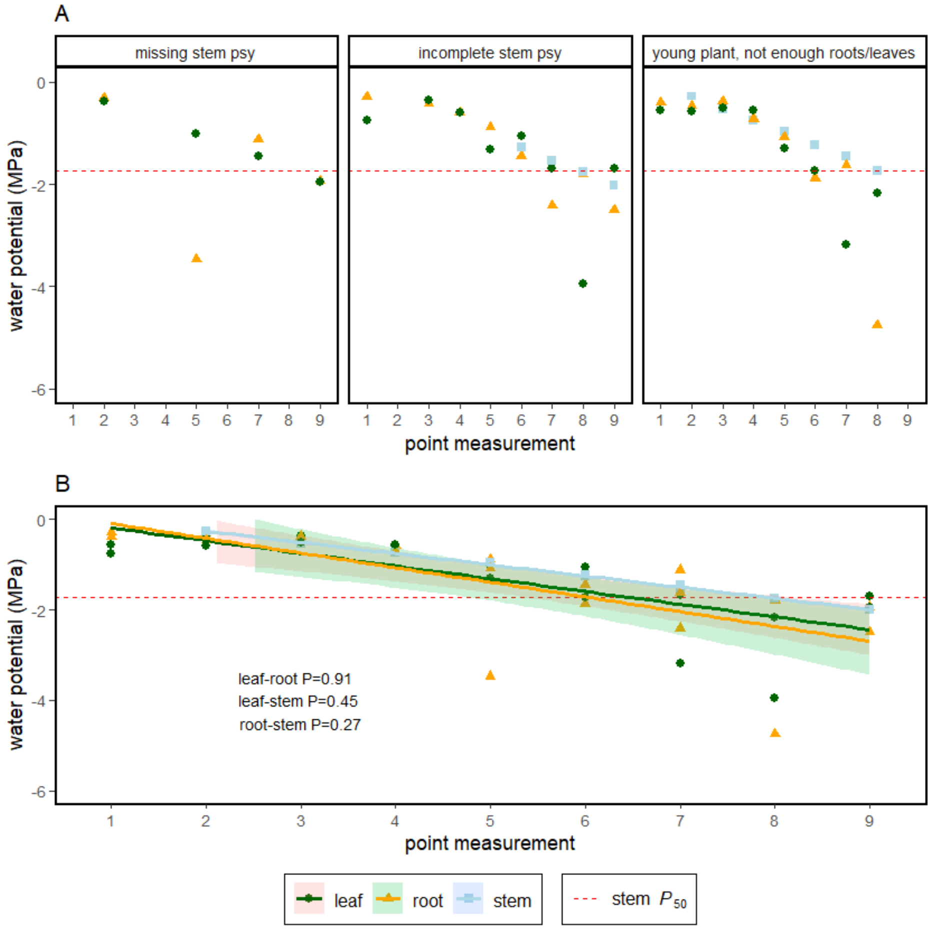

Supplementary Figure S1: Whole-plant water potential: Three extra incomplete replicate plants, with issues with stem WP and plant age. No significant differences observed between organs of the additional plants. Simultaneous water potential measurements of leaves, roots, and stems using psychrometry in three intact soybean (*G. max*) plants to capture each organ at the same thresholds of water stress, showing consistent whole-plant water potential rates of drying. A) Rates of drying in roots, stems, and leaves for the three different plants (1 to 3). B) Changes in water potential of leaves, roots and stems during the dehydration experiment, regressions show means ± SE shading (n=3 plants per water potential measurement), indicating no significant differences between pairwise (ANCOVA-adjusted t-tests) comparisons of mean slopes.

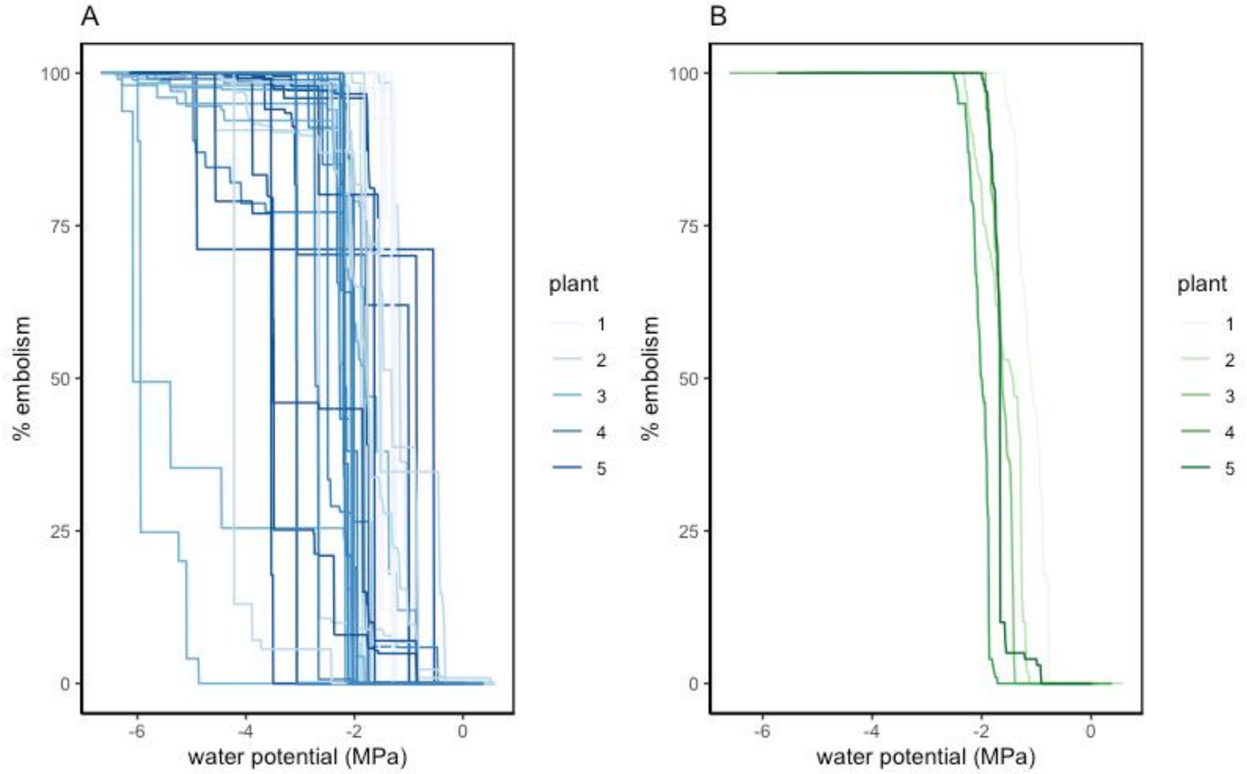

Supplementary Figure S2: Vulnerability of individual fine roots making up the root system (panel A), and leaves (panel B) of the five plants. 28 individual fine roots segments across five plants (A, blue) show notable variability in their vulnerability to embolism compared with leaves (B, green)..

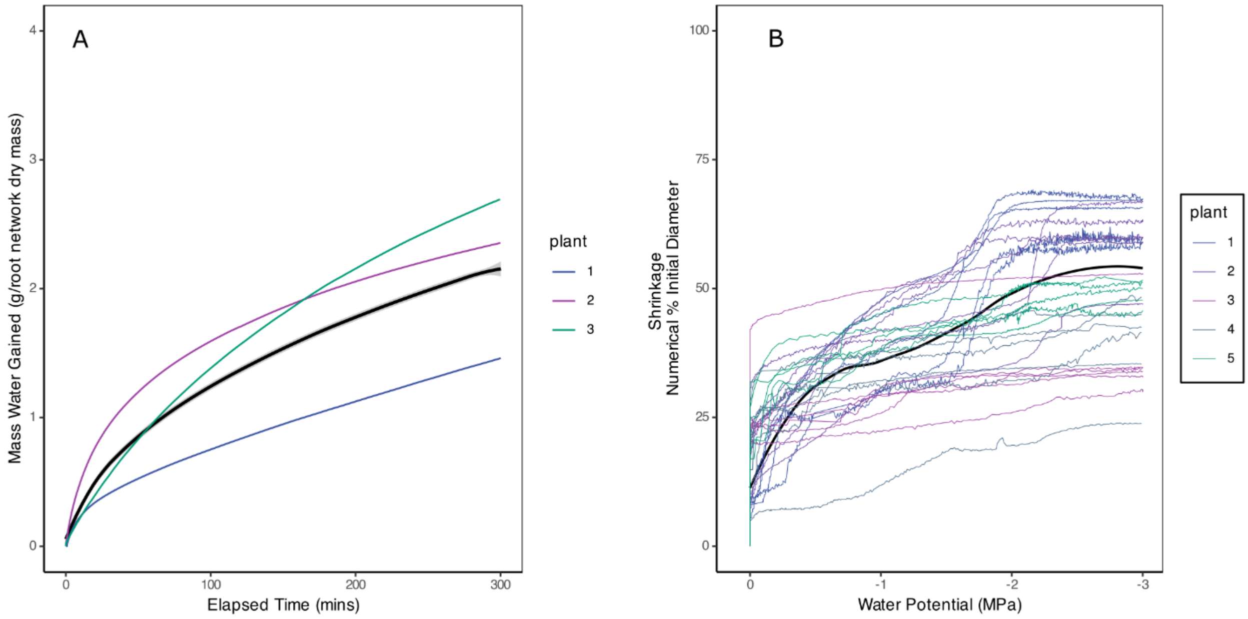

Supplementary Figure S3: Rate of root network capacitive rehydration compared with rate of root shrinkage. Facet A) Mass of water gained by the root networks during capacitance assessment when rehydrated from ~ -0.7 MPa, a threshold known to cause significant damage to cortex but not stele cells (n=3 plants). The rehydration rates of roots reflect (Facet B) non-linear rate of shrinkage seen from the Optical Dendrometry data (n=5 plants, comprising 28 roots). Black lines designate mean rate per facet. Facets coloured by plant rep (separate experimental plants).

Supplementary Tables

Supplementary Table 1: Mean fine root shrinkage from an initial hydrated diameter (100%), and percentage shrunk on a scale of 0% shrinkage at 0MPa to 100% shrinkage at the cessation of embolism.

Threshold	Mean % of Initial Hydrated Diameter	SD Initial Hydrated Diameter	Mean Normalised % of Initial Hydrated Diameter	Mean Normalised % shrunk	SD Normalised
0.00	100	0	100.00	0.00	0.00
-0.10	76.32	8.35	50.92	49.08	19.27
-0.25	72.73	7.93	44.64	55.36	16.95
-0.50	69.06	7.69	38.10	61.90	13.91
-0.75	65.98	8.52	32.27	67.73	13.32
-1.00	63.52	8.74	27.36	72.64	12.54
-1.25	61.70	9.05	23.66	76.34	11.96
-1.50	60.01	9.32	20.30	79.70	11.20
-1.75	56.93	11.37	15.15	84.85	9.54
-2.00	54.60	12.79	11.02	88.98	9.51

Supplementary Table 2: Optical Vulnerability means

organ	P12 Mean	P12 SD	P50 Mean	P50 SD	P88 Mean	P88 SD
leaf	-1.41	0.42	-1.57	0.33	-1.90	0.34
Mean root	-1.29	0.42	-1.80	0.31	-2.58	0.84
stem	-1.36	0.50	-1.74	0.46	-2.09	0.54

Supplementary List 1: Root pressure probe list of publications:

Frensch J, Hsiao TC. 1993. Hydraulic propagation of pressure along immature and mature xylem vessels of roots of Zea mays measured by pressure-probe techniques. *Planta* **190**(2): 263-270.

Itoh K, Nakamura Y, Kawata H, Yamada T, Ohta E, Sakata M. 1987. Effect of osmotic stress on turgor pressure in mung bean root cells. *Plant and Cell Physiology* **28**(6): 987-994.

Jones H, Tomos AD, Leigh RA, Wyn Jones RG. 1983. Water-relation parameters of epidermal and cortical cells in the primary root of Triticum aestivum L. *Planta* **158**: 230-236.

Lee SH, Zwiazek JJ, Chung GC. 2008. Light-induced transpiration alters cell water relations in figleaf gourd (Cucurbita ficifolia) seedlings exposed to low root temperatures. *Physiologia Plantarum* **133**(2): 354-362.

Leigh RA, Tomos AD. 1983. An attempt to use isolated vacuoles to determine the distribution of sodium and potassium in cells of storage roots of red beet (Beta vulgaris L.). *Planta* **159**(5): 469-475.

Lew RR. 1996. Pressure Regulation of the Electrical Properties of Growing Arabidopsis thaliana L. Root Hairs. *Plant Physiology* **112**(3): 1089-1100.

Meuser J, Frensch J. 1998. Hydraulic properties of living late metaxylem and interactions between transpiration and xylem pressure in maize. *Journal Of Experimental Botany* **49**(318): 69-77.

Neumann PM, Azaizeh H, Leon D. 1994. Hardening of root cell walls: a growth inhibitory response to salinity stress. *Plant, Cell & Environment* **17**(3): 303-309.

Pritchard J, Barlow PW, Adam JS, Tomos AD. 1990a. Biophysics of the inhibition of the growth of maize roots by lowered temperature. *Plant Physiology* **93**(1): 222-230.

Pritchard J, Tomos AD, Wyn Jones RG. 1987. Control of Wheat Root Elongation Growth. *Journal Of Experimental Botany* **38**(6): 948-959.

Pritchard J, Williams G, JONES RGW, Tomos A. 1989. Radial turgor pressure profiles in growing and mature zones of wheat roots—a modification of the pressure probe. *Journal Of Experimental Botany* **40**(5): 567-571.

Pritchard J, Wyn Jones RG, Tomos AD. 1988. Control of wheat root growth. The effects of excision on growth, wall rheology and root anatomy. *Planta* **176**(3): 399-405.

Pritchard J, Wyn Jones RG, Tomos AD. 1990b. Measurement of yield threshold and cell wal extensibility of intact wheat roots under different ionic, osmotic and temperature treatments. *Journal Of Experimental Botany* **41**(6): 669-675.

Radin JW, Matthews MA. 1989. Water transport properties of cortical cells in roots of nitrogen- and phosphorus-deficient cotton seedlings. *Plant Physiology* **89**(1): 264-268.

Shabala SN, Lew RR. 2002. Turgor Regulation in Osmotically Stressed Arabidopsis Epidermal Root Cells. Direct Support for the Role of Inorganic Ion Uptake as Revealed by Concurrent Flux and Cell Turgor Measurements. *Plant Physiology* **129**(1): 290-299.

Steudle E, Jeschke W. 1983. Water transport in barley roots: measurements of root pressure and hydraulic conductivity of roots in parallel with turgor and hydraulic conductivity of root cells. *Planta* **158**: 237-248.

Steudle E, Oren R, Schulze E-D. 1987. Water transport in maize roots: measurement of hydraulic conductivity, solute permeability, and of reflection coefficients of excised roots using the root pressure probe. *Plant Physiology* **84**(4): 1220-1232.

Winch S, Pritchard J. 1999. Acid-induced wall loosening is confined to the accelerating region of the root growing zone. *Journal Of Experimental Botany* **50**(338): 1481-1487.

Zimmermann U, Rygol J, Balling A, Klock G, Metzler A, Haase A. 1992. Radial turgor and osmotic pressure profiles in intact and excised roots of Aster tripolium: Pressure probe measurements and nuclear magnetic resonance-imaging analysis. *Plant Physiology* **99**(1): 186-196.
